# Truth, control, and value motivations: the “what,” “how,” and “why” of approach and avoidance

**DOI:** 10.3389/fnsys.2014.00194

**Published:** 2014-10-14

**Authors:** James F. M. Cornwell, Becca Franks, E. Tory Higgins

**Affiliations:** ^1^Department of Psychology, Columbia UniversityNew York, NY, USA; ^2^Animal Welfare Program, University of British ColumbiaVancouver, BC, Canada

**Keywords:** motivation, approach, avoidance, promotion, prevention, truth, control, value

## Abstract

The hedonic principle—the desire to approach pleasure and avoid pain—is frequently presumed to be *the* fundamental principle upon which motivation is built. In the past few decades, researchers have enriched our understanding of how approaching pleasure and avoiding pain differ from each other. However, more recent empirical and theoretical work delineating the principles of motivation in humans and non-human animals has shown that not only can approach/avoidance motivations themselves be further distinguished into promotion approach/avoidance and prevention approach/avoidance, but that approaching pleasure and avoiding pain requires the functioning of additional distinct motivations—the motivation to establish what is real (truth) and the motivation to manage what happens (control). Considering these additional motivations in the context of moral psychology and animal welfare science suggests that these less-examined motives may themselves be fundamental to a comprehensive understanding of motivation, with major implications for the study of the “what,” “how,” and “why” of human and non-human approach and avoidance behavior.

## INTRODUCTION

The hedonic principle has existed for at least as long as we have had the capacity to write down our thoughts about ourselves, being recorded, for example, in the teachings of the ancient Greek philosopher Epicurus. In modern times, the principle reached its fullest expression as a foundation for human psychology and ethics in Bentham’s (1789/2007) influential *An Introduction to the Principles of Morals and Legislation:* “Nature has placed mankind under the governance of two sovereign masters, *pain*, and *pleasure*. It is for them alone to point out what we ought to do, as well as to determine what we shall do” (Bentham, 1789/2007). The principle that humans and other animals approach desired end-states and avoid undesired end-states has served as the foundation for important theories across disciplines from political theory (Neumann and Morgenstern, 1944/2007) to behavioral economics (Simon, 1955). In many ways, our common economic life is built around this basic idea (Smith, 1776/2003), and it is one of the primary assumptions of many theories of animal behavior, both human and non-human (Thorndike, 1911, 1935). The fact that human beings and other animals approach pleasure and avoid pain has been treated as *the* fundamental principle from which all other examinations of motivation must flow (Watson, 1913; Freud, 1920/1950; Skinner, 1938).

The ascendance of approach and avoidance in the psychology literature co-occurred with the rise of behaviorism (Thorndike, 1911; Watson, 1913; Skinner, 1938, 1953). Behaviorist theorists, as a rule, argued that one cannot scientifically reason beyond those actions which can be directly observed and the contingencies that surround those actions—namely reinforcements and punishments (Thorndike, 1911). Any speculation as to the internal workings of those motivations, whether cognitive or otherwise, was eschewed as unscientific (Watson, 1913). The hard scientific work of a number of psychologists led to a cognitive revolution in the middle of the 20th century overthrowing behaviorist assumptions (e.g., White, 1959; Bandura, 1977), yet the premise regarding approach and avoidance as *the* fundamental distinction in motivation has remained (for a review, see Higgins, 1997).

Beginning in the late 20th century, many scientific discoveries were made further distinguishing between approaching positive end-states vs. avoiding negative end-states (Carver and Sheier, 1998; Carver, 2004), and extended this distinction into many additional areas of research. For example, Goal Orientation Theory has made significant contributions to our understanding of achievement motivation (Dweck, 1986; Pintrich, 2000; Elliot, 2005). Although future explorations of truth and control motives may discover interesting relations to achievement motivation, our model of motivation is theoretically orthogonal to these lines of research. For this reason, their possible interrelationships will not be discussed in detail here. For the purposes of this review, we focus on the contributions that have culminated in prominent theoretical advances in other fields dealing with motivation, including moral psychology (e.g., Janoff-Bulman et al., 2009) and animal welfare science (e.g., Fraser and Duncan, 1998). In this paper, we argue that, in part due to its resounding success as a theoretical framework for behavior, the approach/avoidance distinction is now at risk of becoming a “one size fits all” principle. Specifically, multiple kinds of motivations are now treated as if they entailed a simple approach/avoidance hedonic distinction when, in fact, not only are they not simply hedonic, they are also distinct from one another in important ways. We propose that these distinctions matter to future research in many fields of study and that attending to them may yield important breakthroughs.

In this paper, though we argue that this treatment of approach/avoidance as a “one size fits all” principle of motivation is difficult to sustain in light of recent research, we fully recognize the importance of this distinction for motivation science. We thus begin the paper by outlining the ways in which the approach/avoidance distinction has made significant contributions to two areas of study: non-human animal behavior and moral psychology. We then review recent work in these fields demonstrating that other motivational distinctions need to be taken into account as well, and argue that some of these motives may in fact be fundamental to fully understanding the approach/avoidance distinction itself—looking within the “one size fits all” approach/avoidance principle to delineate the “what,” “how,” and “why” of these orientations and behaviors.

## ADVANCES IN APPROACH AND AVOIDANCE MOTIVATION RESEARCH

The hedonic principle has provided psychologists with considerable predictive power since the foundational days of psychology in the 19th century. This includes research on non-human animals—driven by an understanding that some (though not all, see Higgins and Pittman, 2008) of the major components of human nature are shared with non-human animals through the branching process of evolution (Watson, 1913; Skinner, 1938, 1953)—and the search for essential components of human nature around which we can organize an ethics to guide our common life (Mill, 1863/2007; for a recent review, see Kitcher, 2011). Interestingly, though assumptions about approach and avoidance have underpinned the study of animal behavior and morality for over 100 years, some particular advances in each field have only been achieved by distinguishing between approach and avoidance in recent decades.

### APPROACH/AVOIDANCE IN NON-HUMAN ANIMALS

For many, the appeal of the study of non-human animals lies in the ability of this line of inquiry to reveal fundamental truths about nature. Evolutionary theory in particular has shown us that human beings constitute only one species of animal, and that we are, at our most basic, mammals subject to many similar kinds of motivations as other animals. Approach and avoidance motivations have played a substantial part in understanding non-human animal behavior. For example, approach and avoidance orientations have been tied to underlying individual differences between animals. Certain animals, more than others, are willing to take risks in order to achieve their goals, while other animals, more than others, are consistently more risk-averse in their behavior (Wilson et al., 1994). Animals that fall into the former category have been classified as “bold,” whereas those that fall into the latter category have been classified as “shy.” These individual differences map onto a greater reliance on approach and avoidance orientations, respectively.

The bold/shy continuum has received an enormous amount of attention in recent years. Researchers have found individual differences on this continuum with attendant behavioral implications in squid (Sinn et al., 2008), fish (Toms et al., 2010), and lizards (López et al., 2005), just to name a few. Their underlying neurological differences have been studied (Reddon and Hurd, 2009), the impact of environmental context in their variation has been researched (Wilson et al., 1993), and the evolutionary origins of these individual differences have been explored (Wilson et al., 1994). Differences along this continuum have been linked to fundamental differences in stress responses (Oswald et al., 2012) and learning (Sneddon, 2003). It is clear that examining the difference in approach and avoidance inclinations, here understood as having either a bold or shy personality, has proven a to be a productive theoretical foundation for scientific exploration of non-human animals. However, as we will describe in more detail later, there are animal behaviors that cannot be understood from just a “one size fits all” approach/avoidance perspective. A more complete picture of non-human animal motivation requires the consideration of additional motivational distinctions.

### APPROACH/AVOIDANCE IN MORAL PSYCHOLOGY

Approach and avoidance motivations are fundamental in another way as well. They can help us to understand one of the most basic principles around which human societies organize themselves: ethics or morality. The principles of approach and avoidance have been integral to the study of ethics since the empiricists of the Scottish Enlightenment designated morality as an instrumental means for bringing about general happiness. For example, Hume (1751/1998), following Hutchinson (1725/2005), argued for the importance of positive and negative sentiments in morality, particularly in moral motivation. Mill (1863/2007) went so far as to argue that pleasure and pain provided a framework both for understanding and predicting human behavior and also for building a system of ethical dos and don’ts: things to approach and things to avoid.

Over the years, however, the predictive power of this hedonic approach has been called into question, perhaps most prominently by Kahneman and Tversky (1979), whose work revealed important differences between what happens psychologically when people approach positive outcomes vs. avoid negative outcomes. In Prospect Theory, they argued that the motive to avoid pain “looms larger” than the motive to attain pleasure, and, importantly, people are relatively risk-seeking when avoiding negative outcomes (in the domain of losses) but are relatively risk-averse when approaching positive outcomes (in the domain of gains). In a manner similar to Prospect Theory’s revisions of the nature of approach vs. avoidance in decision making, Janoff-Bulman et al. (2009) have shown that the two are different with respect to ethical systems. One system, the *proscriptive* system, motivates behavioral inhibition—*avoiding* moral wrongs. The other system, the *prescriptive* system, motivates behavioral activation—*approaching* moral rights. Their research has shown that approach and avoidance are not merely inverted images of one another, but that each has unique goals and tendencies and characteristics associated with it (including differences in political ideology; see Janoff-Bulman et al., 2008). Researchers using this paradigm have theorized important differences between the two systems that could have importance for research on ethics generally (Janoff-Bulman and Carnes, 2013).

These theories are important because they can aid in further understanding the nature of decision making and behavior in human society. However, there are additional motivational distinctions that need to be considered in order to have a more complete picture of ethics, and, as we will see, to understand animal behavior as well. In the next section, we distinguish between two distinct motivational systems around which approach and avoidance motivations are organized, and show how they have made important contributions to the areas of animal welfare research and moral psychology.

## PROMOTION AND PREVENTION VALUE MOTIVES: THE “WHY” OF APPROACH AND AVOIDANCE

**FIGURE 1:**
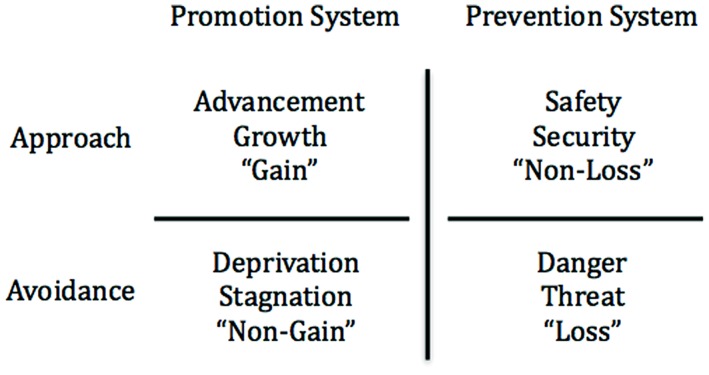
**Promotion and prevention goals within the approach and avoidance systems**.

Over the past two decades, research on approach and avoidance has been qualified by research on regulatory focus theory (Higgins, 1997, 1998). According to regulatory focus theory, the valued goals of approaching desired end-states and avoiding undesired end-states are organized into two independent and distinct motivational systems (Molden et al., 2008; Higgins, 2014). The *promotion* system approaches end-states related to nurturance, advancement, and growth while avoiding deprivation or stagnation—the motivation is to advance from the status quo “0” to a better state “+1.” Success or pleasure in promotion is attaining a “+1” (gain) and failure or pain is not attaining a “+1” (non-gain). The *prevention* system approaches end-states related to security and safety while avoiding danger or threat—the motivation is to maintain a satisfactory status quo “0” against a worse state “-1.” Success or pleasure in prevention is maintaining “0” (a non-loss) and failure or pain is not maintaining “0” (a loss). A visual representation of these systems with respect to the approach and avoidance systems is available in **Figure 1**.

We should note a few other relevant distinctions within regulatory focus theory (for a fuller account, see Scholer and Higgins, 2008). Both when approaching desired end-states and avoiding undesired end-states, humans and non-human animals can pursue their goal with either an eager strategy of seeking opportunities to make progress or a vigilant strategy of being careful and avoiding mistakes. Whether approaching desired end-states or avoiding undesired end-states, the preferred strategy (i.e., what fits) for those with a promotion focus is an eager strategy whereas the preferred strategy for those with a prevention focus is a vigilant strategy (Higgins, 2000). But, notably, the non-preferred strategy (i.e., a non-fit) will also be used, sometimes because it is dictated by the situation (e.g., instructions about what strategy to use, like a team leader telling other team members to “be careful”). Finally, it should also be noted that either taking action (behavioral approach) or inhibiting action (behavioral avoidance) can occur in the service of strategic eagerness or in the service of strategic vigilance. For example, as we will see below, an animal concerned about safety (prevention) might be careful (vigilant strategy) to approach something new in the cage (behavioral approach) in order to make sure that it is safe, or approach a noxious object in the cage in order to bury it (behavioral approach).

The literature has seen a proliferation of research based on this model showing that the promotion and prevention distinction has significant explanatory power independent of the hedonic approach and avoidance motivations (Higgins et al., 1997, 2003; Förster et al., 1998, 2001; Malaviya and Brendl, 2014). This research has covered a variety of domains from persuasion (Cesario et al., 2004), to negotiation (Appelt et al., 2009), to consumer choice (Higgins, 2002), to interpersonal relationships (Molden et al., 2009). Theoretically, it has inspired more comprehensive models of the approach and avoidance motives, understanding them as being further subdivided into promotion goals (including “ideal” hopes and aspirations) on the one hand and prevention goals (including “ought” duties and obligations) on the other (Crowe and Higgins, 1997; Higgins, 1997). Recently, there is even evidence in neuroscience that the two kinds of distinctions—promotion vs. prevention in contrast to approach vs. avoidance—are associated with independent patterns of brain activation (Eddington et al., 2007; Strauman and Wilson, 2010). In this section, we will be focusing on new research applied to the fields of animal welfare science and moral psychology to highlight the fundamental character of the promotion vs. prevention distinction.

### PROMOTION/PREVENTION IN NON-HUMAN ANIMALS

The promotion vs. prevention distinction is well-established in humans, but recent research supports their existence in non-human animals as well. For example, one set of studies examined individual differences in behaviors reflecting promotion and prevention motivations among a group of zoo-housed cotton-top tamarins (Franks et al., 2013b). Through extensive observation, researchers identified individuals who consistently prioritized gains over safety (time spent eating in the open) or safety over gains (time spent hiding) in order to classify them as more promotion-focused or prevention-focused, respectively. Importantly, an approach/avoidance model of behavior (using the bold/shy conceptualization mentioned above) could similarly classify the above individuals as being more approach-oriented (“bold”) or avoidance-oriented (“shy”), respectively. Thus, it is unclear whether the monkeys appearing to prioritize safety are *avoiding* danger—which would be more in line with an avoidance-motivated model of behavior—or *approaching* security—which would be more in line with a prevention-motivated model of behavior. Similarly, it is unclear whether the monkeys appearing to prioritize gains are approaching *indiscriminately*—which would be more in line with an approach-motivated model of behavior—or are motivated by gains *specifically*—which would be more in line with a promotion-motivated model of behavior.

In order to test which model—approach/avoidance vs. promotion/prevention—made a better account of the animals’ behavior, researchers placed two different kinds of novel enrichment items into the monkey’s housing: one a “gain” enrichment and the other a “non-gain” enrichment (Franks et al., 2013b). If the primary difference separating the individuals described above was an approach/avoidance difference, then those classified as “approach”-oriented should approach *all* the novel objects more quickly than those classified as “avoidance”-oriented since coming into the open to examine a novel stimulus carries risk and should be unconditionally more aversive to the “avoidance”-oriented individuals than the “approach”-oriented individuals. However, if instead the two types of monkeys were actually different according to the promotion/prevention distinction, then this dynamic should only be *conditionally* true in the case of the “gain” object. In the case of the “non-gain” object, a regulatory focus model of behavior suggests that a promotion-focused individual should be uninterested because it has no gain potential, while the prevention-focused individual should be interested in cautiously examining the object in order to establish its status as a non-threat. The results confirmed the regulatory focus distinction, with prevention-focused monkey *approaching* a novel “non-gain” object *faster* than promotion-focused monkey (Franks et al., 2013b).

Research with rats has also shown that there are individual differences with respect to preferences for promotion vs. prevention goals. In one study, these goals were operationalized as time spent near a location containing food reward (promotion) vs. time spent near a location that turned the overhead light off, which, because they are nocturnal, creates security for rats (prevention; Franks et al., 2012). Though the promotion or prevention behavior of individual rats was stable in this test over several weeks, once again, from these behaviors alone it is unclear whether the observed differences are due to an approach/avoidance distinction or a promotion/prevention distinction. Were the darkness-preferring rats *avoiding* light as aversive or *approaching* darkness to maintain security? Were the treat-preferring rats approaching indiscriminately or approaching gains specifically?

To test these alternative hypotheses, the rats were observed in a different apparatus into which a noxious stimulus was introduced (Franks et al., 2012). If the rats preferring darkness were driven primarily by an avoidance orientation, then we would expect them to move as far away from the noxious stimulus as possible. If, however, the rats preferring darkness were driven by a prevention orientation, then we would expect them to *approach* the noxious stimulus in order to bury it, which is a rat’s natural defensive behavior and means by which to restore safety (Pinel and Treit, 1978). The results favored the latter hypothesis: time with the noxious stimulus to bury it (vigilant behavioral approach in the service of prevention) was predicted by time spent maintaining darkness (prevention) and not by time spent with food reward (promotion). This animal behavior research is in line with recent work in humans examining how a prevention focus can actually motivate riskier behavior (i.e., more approach-oriented behavior) when under conditions of loss or threat (Scholer et al., 2010).

Finally, another set of studies with a separate group of rats further distinguished promotion and prevention motivation from simple approach/avoidance motivation (Franks et al., 2014). Rats were again placed in an environment in which they could focus on maximizing gains (obtaining treats) and on maintaining safety (keeping the room dark) and again stable individual differences in promotion and prevention motivation were observed. In this experiment, however, researchers were able to collect a measure of chronic stress (or poor welfare) and found it to be inversely related to *both* promotion and prevention. This finding parallels empirical work in humans (Grant and Higgins, 2003) and corresponds to regulatory focus theory, which predicts positive emotions and well-being resulting from being effective at both promotion and prevention goals (Higgins, 1997). This finding would be somewhat puzzling from a simple approach/avoidance model of motivation and welfare, however, because if avoidance was driving the prevention animals’ behavior, they should be more fearful than other animals and thus have lower welfare.

In sum, the behavior in non-human animals suggests a distinction between promotion goals and prevention goals *within* the approach and avoidance systems. Indeed, given its presence among non-human animals, even those as evolutionarily distant from humans as rats, this regulatory focus distinction—the “why” of approach and avoidance—may turn out to be a fundamental characteristic within the approach/avoidance distinction. This distinction is not limited to non-human animal welfare, however. As we discuss next, the role of promotion and prevention in moral psychology has been theoretically and empirically demonstrated as well.

### PROMOTION/PREVENTION IN MORAL PSYCHOLOGY

Over the past several years, there has been a push to examine the approach and avoidance distinction within moral psychology. However, until very recently, the independent role of promotion goals and prevention goals in the area of ethics has been largely unexplored. As with the non-human animal research outlined above, goals to approach moral behavior can be understood as stemming from either ideals or oughts, and efforts taken to avoid immoral behavior can similarly be understood as avoiding discrepancies with these distinct kinds of goals.

The first major exploration of promotion and prevention motivations in morality was carried out by Camacho et al. (2003). In a series of studies, these researchers found that different strategic framing of moral errors (“sins”) created more intense feelings of regret depending on whether a person had a strong promotion or prevention focus. For example, those whose sins involved an error of *commission* (not being vigilant enough to avoid doing something bad) experienced more regret when they had a stronger prevention focus, whereas those whose sins involved an error of *omission* (not being eager enough to do something good) experienced more regret when they had a stronger promotion focus.

Like the animal behavior studies above, it would be possible to make similar predictions as those above relying only on the approach/avoidance distinction, instead stating that those who experience their errors of commission as more wrong are more motivated by avoidance inclinations and those who experience their errors of omission as more wrong are more motivated by approach inclinations. Are those with stronger promotion goals simply more concerned about approaching moral rights? Similarly, are those with stronger prevention goals simply more concerned about avoiding moral wrongs?

Other results from the Camacho et al. (2003) research provide evidence that the regulatory focus distinction matters for ethical responses beyond simple approach/avoidance. If the effects were due entirely to concern with approaching moral rights or avoiding moral wrongs, then the specific content of those rights and wrongs would not matter. However, they did matter significantly. Consistent with the regulatory focus perspective, those with a strong prevention focus experienced failures of social responsibility (a prevention security concern) as more wrong than those with a weak prevention focus (and those with strong promotion focus), whereas those with a strong promotion focus experienced failures to support others (a promotion nurturance concern) as more wrong than those with a weak promotion focus (and those with a strong prevention focus). Importantly, this effect was independent of whether the wrong was framed as an act of commission or omission.

In another study by Camacho et al. (2003), promotion and prevention had important effects with respect to positive moral evaluations as well. In this case, the difference between approach/avoidance on the one hand and regulatory focus on the other was made even more apparent. This study related participants’ judgments of the perceived appropriateness of a conflict resolution *strategy* that was taken by his or her parent or guardian (“We ask that you think back to a time when you had a conflict with your parent or guardian and he/she resolved the conflict by….”). The strategic fit with promotion or prevention was *independent* of whether the strategy involved an attempt by the parent or guardian to increase future good behaviors (approach) or to decrease future bad behaviors (avoidance). The strategy could either be eager/positive (e.g., encouragement to succeed, providing opportunity), eager/negative (e.g., taking away a privilege, acting disappointed), vigilant/positive (e.g., safeguarding against undesired behaviors, alerting to potential dangers), and vigilant/negative (e.g., raising his/her voice, providing criticism).

If it were the case that the omission/commission difference associated with promotion/prevention was actually an effect of approach/avoidance, then those with a stronger promotion focus should see the positive approach resolutions as more appropriate than the negative avoidance resolutions, and those with a stronger prevention focus should see the negative avoidance resolutions as more appropriate than the positive approach resolutions. This pattern was not found. Instead, the researchers found that those with a stronger promotion focus preferred the eager resolutions over the vigilant resolutions regardless of whether the resolutions involved positive approach or negative avoidance. Similarly, those with a stronger prevention focus preferred the vigilant resolution over the eager resolution, regardless of whether the resolution involved positive approach or negative avoidance (Camacho et al., 2003).

Not only does this empirical evidence support the importance of the distinction between promotion and prevention independent of approach/avoidance in the area of ethics, more recent research indicates that recognizing this difference can help us to better understand some of the more well-known conundrums within moral psychology. For example, research on the promotion and prevention systems has shown that those processing in a promotion focus tend to make judgments and decisions based on feelings, while those processing in a prevention system tend to make judgments and decisions based on reasons (Pham and Avnet, 2004; Avnet and Higgins, 2006). The divide between feelings and reasons is analogous to the well-known division within moral psychology between social-intuitionists on the one hand who understand moral judgments and decisions as primarily arising from intuitions or affect (Haidt, 2001), and cognitive-developmental theorists on the other who approach morality as a fundamentally cognitive enterprise (Kohlberg, 1969). An intriguing possibility is that they may both be correct: processing moral goals in a promotion manner may rely on intuitions/affect, while processing moral goals in a prevention manner may rely on cognition/reasons.

So far the evidence has supported this hypothesis. Consider, for example, the infamous “incest” scenario put forward by social-intuitionists as a prototypical scenario evoking a negative intuitive response but a more muted deliberative response (Haidt et al., 1993). In this scenario, a brother and sister sleep together once, but not before taking precautions to avoid any negative consequences of the act (i.e., using two forms of contraception, agreeing to keep it a secret). Recent research has found that those who process this scenario in a promotion focus see the incest as more wrong than those who process it in a prevention focus (Cornwell and Higgins, unpublished manuscript). Importantly, in another study, Cornwell and Higgins (unpublished manuscript) examined the promotion/prevention distinction independent of the approach/avoidance distinction. In this study, participants were experimentally divided into four groups: promotion/approach, promotion/avoidance, prevention/approach, and prevention/avoidance. This study replicated the “stronger moral condemnation for promotion than prevention” effect for the incest scenario while also demonstrating the effect for another scenario (the equally infamous “dog-eating family” scenario, see Haidt et al., 1993) but, importantly, showed no effect of the approach vs. avoidance distinction (Cornwell and Higgins, unpublished manuscript). This research provides another example of how understanding distinct types of avoidance motives—the promotion version and the prevention version—can contribute to a better understanding of how moral judgments can have very different motivational underpinnings.

Another area in which the distinction between the promotion and prevention systems has proved relevant in ethics is in the examination of multiple moral actions across time. Much research has been devoted to understanding how different ways of framing moral goals can influence people to behave in either consistently good ways or to switch from good to bad (e.g., engaging in bad behavior because prior good behavior has “licensed” you to do so; Sachdeva et al., 2009). For example, in one study, when participants purchased “green” environmentally friendly products, compared to those who purchased conventional products, they later behaved less altruistically and were more likely to cheat and steal on subsequent tasks (Mazar and Zhong, 2010). These latter immoral behaviors (i.e., cheating and stealing) were understood as “licensed” by prior moral behaviors (i.e., the purchase of a “green” product). However, in spite of research showing this effect, there are conditions in which behaving morally can actually lead to moral consistency rather than moral licensing (Conway and Peetz, 2012), and thus many questions surrounding these phenomena remain (Merritt et al., 2010). The promotion/prevention distinction provides some insight into the motivational underpinnings of these effects.

As noted above, research has shown that when people process decisions within the prevention system, they tend to use vigilant means to pursue stability and security, which leads them to favor the *status quo* options over alternatives (Chernev, 2004). One example of the prevention focus leading to a tendency toward preserving the status quo is the research finding that the endowment effect is stronger for individuals with a prevention focus than a promotion focus (Liberman et al., 1999). Another example is the propensity of prevention-focused individuals (but not promotion-focused individuals) to repeat in the present ways of doing things that they experienced in the past. Studies have shown that prevention-focused managers manage others with the style that they received when they were managed in the past, and that they preserve this status quo even when they disliked receiving this style of management (Zhang et al., 2011). The extension to moral psychology would predict that, compared to those who are more promotion-focused and those with a weak prevention focus, those with a strong prevention focus should be more likely to behave in a consistent manner, regardless of whether their original behavior was moral or immoral.

This prediction was supported in recent research. Participants with a strong prevention focus (whether measured chronically or experimentally induced) were more likely to repeat past good behaviors or repeat past bad behaviors compared to those who were either low in chronic prevention or induced into a promotion focus (Zhang et al., 2014). Importantly for the purposes of this paper, these effects occurred whether or not the initial moral behavior involved doing something bad, that is, an *immoral* act that ought to be *avoided*, such as cheating, or involved failing to do something good, that is, a *moral* act that ought to be *approached*, such as pledging money to a charity. This research thus provides a further demonstration of the importance of the promotion/prevention distinction within approach and within avoidance in the domain of ethics.

In sum, we see that the promotion/prevention distinction provides us with a more comprehensive view of the “why” of approach and avoidance. That is, when considering approach and avoidance as “why” we are motivated to do things, it is necessary to go beyond this simple distinction to recognize that there are two fundamentally different value systems within approach and within avoidance. Approaching desired end-states and avoiding undesired end-states in a promotion focus is fundamentally different from approaching desired end-states and avoiding undesired end-states in a prevention focus. Therefore, to understand how the approach value system works it is necessary to know how it works in promotion *and* how it works in prevention. Similarly, to understand how the avoidance value system works it is necessary to know how it works in promotion *and* how it works in prevention.

But what of the other two questions we seek to answer: the “what” and the “how” of approach/avoidance motivation? In the next section, we provide an overview of the importance of these two questions for understanding approach and avoidance behaviors and provide evidence that they are as important to understand as value (promotion and prevention) and cannot be understood in terms of the approach/avoidance distinction alone.

## TRUTH AND CONTROL MOTIVES: THE “WHAT” AND “HOW” OF APPROACH AND AVOIDANCE

While the distinction between prevention vs. promotion goals is critical for advancing our understanding of how the approach and avoidance systems work, it is also important to examine what approach/avoidance presupposes. The study of humans and other animals tends to be motivated by the question of why people and animals behave the way they do, and approach and avoidance motives (further distinguished by promotion and prevention goals) address this by providing an answer (value from having desired results). But asking “why” of an action presupposes two additional things: it assumes that the action is bringing something about, and it assumes that the action is motivated by some understanding that the animal has of itself and its relation to its environment. The “bringing something about” (or managing to have an effect) constitutes the “how” of the behavior—*control motivation*. The understanding (or establishing what’s real) constitutes the “what” of behavior—*truth motivation*. Both control and truth need to be successful in order for the “why” to be successful. That is, control and truth need to *work together* with value for effective goal pursuit (Higgins, 2012). As such, it is not enough to consider approach/avoidance value alone, even when the promotion/prevention distinction is included in this consideration. In order to demonstrate the fundamental nature and independence of truth and control in goal pursuit, we will once again examine them in the context of the same two domains—motivation in non-human animals and moral motivation in humans.

### TRUTH AND CONTROL IN NON-HUMAN ANIMALS

Non-human animals do indeed approach valued end-states and avoid aversive end-states, but in order to effectively do so, they need to *learn* the contingencies of those end-states and actively *adapt* to their environment so that those ends might be achieved. That is, animals need to understand the contingencies and characteristics of their environments (truth) and take an active part in managing that environment (control) in order to bring about valued outcomes like security (prevention) and growth (promotion).

First, the “what” question is aimed at discovering the relevant ways in which the world works. Answering this question requires exploration and learning, both of which have been observed in non-human animals, even when no valued outcome is present or attainable from them (Franks and Higgins, 2012). One of the earliest researchers to document observations of this tendency in non-human animals was Harlow (1950) who presented a group of rhesus macaques with a complex mechanical puzzle. The monkeys engaged with the puzzle in order to understand how it worked, in spite of the fact that they received no reward for doing so—in fact, adding a food reward to the task tended to disrupt learning rather than facilitate it (Harlow et al., 1950). This study suggested that the “what” question is inherently motivating independent of “why” valued outcomes. More recently, animal welfare scientists have shown that animals are motivated to explore and learn—aspects of the “what” question and truth motivation in general. For example, rats will give up known reward and incur risk in order to explore novel environments (Franks et al., 2013a) and goats will interact with a learning device in order to obtain sips of water, even when water is available through less arduous means (Langbein et al., 2009).

Furthermore, we can see how this “what” question is essential to the example involving individual differences in cotton-top tamarins’ promotion and prevention focus described earlier (Franks et al., 2013b). As mentioned above, different animals were motivated to approach different stimuli in their environment with different response latencies depending on whether they were more motivated to approach security or approach gains. However, the example also involved the ability to *discriminate* between “gain” and “non-gain” stimuli, presupposing the motivation to learn that distinction. The monkeys were able to classify the stimuli as belonging to a particular self-regulatory category in order for them to respond to it in accordance with their regulatory focus orientation. Put another way, their ability to assess the “what” of the stimuli enabled their action in accordance with their preferred “why” (i.e., valued outcomes).

Second, animals also need to answer the “how” question—how to control or manage the situation in order to actually achieve the valued promotion or prevention, approach or avoidance goal. Animal welfare science has been instrumental in showing that the control or management activities themselves, such as the act of building a nest or pushing a lever to obtain food, are motivating independent of the actual result achieved. For example, Carder and Berkowitz (1970) describe the case of rats who could effortlessly attain food from a free food dish in front of them, but instead push the food dish out of the way in order to press a lever to make a pellet of the same food fall into the food tray (for a general review of “contrafreeloading,” see Osborne, 1977). In other words, beyond the desire to simply have good outcomes and the absence of bad outcomes, animals are also motivated to take an active part in bringing about these valuable results, even when doing so unnecessarily expends energy or involves risks (Franks and Higgins, 2012).

This control motivation is also apparent in the example involving the promotion-focused and prevention-focused rats cited in the previous section, particularly in the case of the prevention-focused rats (Franks et al., 2012). As mentioned above, some of the rats in the study were prevention-focused in the sense that they focused their energy on safety through maintaining darkness. When an aversive stimulus was placed within their environment, however, these same prevention rats were also the most approach-oriented. In contrast to the interpretation based on the theoretical construct of approach/avoidance, in which animals are motivated to minimize pain and maximize pleasure, some of the rats in this experiment are actually *intensifying* an avoidable aversive experience (Franks et al., 2012). One way of making sense of this seeming inconsistency is to posit a distinct motivation to act upon the environment independent of immediate pleasurable or aversive experiences. Thus the rats in the experiment not only established the “what” of the stimulus (i.e., classify it as a threat), they were also motivated to act upon the environment, to take control of the situation, despite immediate costs. Put another way, their willingness to “pay” for managing their environment suggests the worth they placed on control, on the “how” of approach/avoidance.

The desire to achieve this “how” or control motivation has been observed in a number of instances involving non-human animals (for a more extensive review, see Franks and Higgins, 2012). For example, monkeys have been found to forgo the opportunity to receive their favorite treat in order to be able to choose from a variety of foods, many of which they dislike (Addessi et al., 2010). This trade-off suggests that having greater choice, and thus greater opportunity to manage the environment, is more motivating to the monkeys than a favorite outcome. Similarly, animal welfare scientists have long recognized that animals often prefer to engage in the activities that lead to desirable goals over simply receiving the goal without having the opportunity to manage the means by which it is achieved (Fraser and Nicol, 2011).

Interestingly, the motivation to actively manage one’s environment has recently been posited as an important individual difference among chimpanzees. Analyses revealed that those animals who engage in the most task-switching—those animals who make the most active changes in managing the environment—are also the same individuals who engage in the most reconciliatory behavior following conflicts, a behavior that involves a great deal of monitoring and control (Webb et al., 2014). Thus the “how” motivation (control) is not only an integral and basic part of approach/avoidance goal pursuit, it is an independent motivation deserving of study in its own right.

These examples show that approaching desired end-states and avoiding undesired end-states presuppose that the individual *understands* its environment (truth) and is motivated to engage in the actions that *bring about* the goal (control). To provide evidence of their distinct importance, we have highlighted cases where these truth and control motivations can be observed independent of value motivations, but we appreciate that these cases are atypical. More often, truth and control motivations integrate with approach and avoidance motivations to create an effective whole—a topic we will discuss in more detail below. But, notably, in these typical cases it is still not approach and avoidance goals working alone. Rather, they are working together with, and depend on, the truth and control motivations. The presence of these distinct motivations in non-human animals points to their fundamental importance.

### TRUTH AND CONTROL IN MORAL PSYCHOLOGY

In the recent modern era, valued outcomes have been argued to be the most fundamental feature of human motivation, and therefore what systems of ethics should concern themselves with most (e.g., Bentham, 1789/2007; Harris, 2010). However, this emphasis on outcomes appears to be an historical and cultural aberration. For example, Aristotle (2009) writes that happiness is constituted by a life of virtue in his *Nicomachean Ethics*, where contemplation (truth) is of paramount importance. Jesus of Nazareth admonishes us to put the “Kingdom of God” and God’s “righteousness” prior to the acquisition of worldly possessions in the Gospel of Matthew (Thomas, 1997). The Buddha remarks that the essential qualities for a noble individual to attain are ethics and wisdom, explicitly setting aside things like high birth and wealth (Walshe, 1995). These thinkers and religious leaders tend to argue that ethics is bound up with motives *other than value*.

The Buddha’s exhortation is particularly instructive in terms of which capacities he treats as ultimately noble: wisdom and ethics. “Wisdom” in this and related cases among ancient philosophers and religious thinkers is generally understood as a virtue like prudence, “good sense,” or the ability to see and know how to respond to situations *as they really are* (i.e., truth; e.g., Aristotle, 2009). “Ethics,” as it was understood by the Buddha and other these ancient thinkers, involves *control over the self* in order that the right actions are carried out effectively in the face of temptations like selfishness, fear, or self-indulgence. The resulting virtues make up the means by which an individual establishes what is real (truth) and manages what happens (control); frequently separated into “intellectual” and “moral” virtues, respectively (e.g., Aquinas, 1981/1274).

Thus, many systems of ethics see the settling on the *correct* courses of action and the *controlled* training of one’s desires to be in line with those courses as *more fundamental* to becoming a good person than designing ethical systems to maximize valued outcomes. Questions of “what” and “how” matter critically for questions of morality. But this theoretical foundation does not exist only within philosophy and religion. By relating research on motivation to research in moral psychology, we can find empirical evidence for the conclusion that these two motives—truth and control—are fundamental to ethics.

One of the earliest questions psychologists asked about morality is how children come to understand what is right and what is wrong (Piaget, 1932/2008). It is not enough, for example, for pain to be *aversive*; children need to learn that pain is *bad or wrong*, and that causing it (to others and to the self) without good reason is *immoral*. In other words, children need to learn that actions and consequences can be right or wrong, good or bad, independent of mere subjective experience. Moral beliefs have an “objectivity” above subjective judgments like taste (Goodwin and Darley, 2008). How is this objectivity achieved?

One psychological mechanism that may shed light on the question of how moral understanding moves from subjective to objective is shared reality. According to the theory of shared reality (Hardin and Higgins, 1996; Echterhoff et al., 2009), human beings achieve a sense of objectivity from their subjective states when they perceive them as verified or shared by a trusted other. With respect to morality, human children observe the reactions of their parents toward particular behaviors, and toward statements about ethical truths, and then emulate within themselves (i.e., share) what they infer to be the perceived inner states (e.g., feelings, beliefs, goals) that underlie those reactions. In a sense, this formulation is an extension of observational learning (Bandura, 1977), but with shared reality it is the inner states that are imitated rather than just the observable behaviors. This is much like the “meta-motivational” self-regulatory factor in achievement motivation, where individuals not only adopt the cognitions appropriate for achievement, but the goals (in our parlance, the “right” value motives) as well (Boekaerts, 1997).

Consider the following research with undergraduate participants for an example of how this process plays out regarding moral beliefs. In a recent study based on Asch’s (1956) classic experiment on the effects of social influence on perception, participants adjusted their moral judgments to be in accord with those around them. In the study, participants were presented with ten moral and amoral behaviors (e.g., murder; telling a friend she doesn’t look fat even though it’s a lie) and asked to declare them either “morally acceptable” or “morally unacceptable.” Together with the participants were four other “participants” who were actually confederates. These confederates were given directions to provide the “right” judgment on some trials and the “wrong” judgment on the “critical” trials that tested whether the participants would be influenced by the confederates to give the “wrong” judgment (i.e., moral judgments opposite to those determined to be the nearly unanimous—at least 97% agreement—private judgments in a pilot study; see Jago et al., 2014). On the “critical” trials in the main study, participants were significantly influenced to agree with the “wrong” judgment that was made by the confederates compared to responses in non-critical trials.

Importantly, these effects appear to be related to the epistemic characteristics of the behaviors in question. For example, one of the behaviors was “murder,” and participants rated the judgment of that behavior as being relatively more “obvious” as being “morally unacceptable” than some of the other scenarios. A good example of a less obvious case of being “morally unacceptable” was one in which a friend tells another that she does not look fat in a pair of jeans even though she does. This scenario is morally ambiguous because, on the one hand, participants could argue that people should value honesty above anything else, and tell the truth even when it hurts a friend’s feelings, but, on the other hand, people are often expected to set the truth aside in order to preserve the feelings of their friends. Thus, it is not surprising that the murder scenario was rated as more morally “obvious” than the lying scenario.

Interestingly for our purposes, if participants are, in fact, motivated to come to the *correct* judgment of the behaviors (truth), they should be more influenced by the unanimous majority opinion when those behaviors are less “obvious” because of greater uncertainty about what is the truth. Consistent with this prediction, the likelihood of providing judgments that agreed with the unanimous majority on “critical” trials significantly increased as the rated “obviousness” of the moral behaviors decreased. That is, it was when behaviors were more morally ambiguous or unclear that participants were more willing to adopt the views of the unanimous majority. They did not simply “go along to get along” or they would have been influenced on all of the “critical” trials equally regardless of how “obvious” the case was. Finally, there was also evidence that when the participants subsequently made private judgments they not only maintained their group-influenced judgments, but also provided rational justifications for their judgments, which is consistent with their continuing in private to believe that they adopted as the truth. These findings support the conclusion that there is an independent motivation to arrive at the moral truth and shared reality is one way to achieve this.

Although establishing what is real is essential in goal pursuit, it is not enough by itself. Individuals are also motivated to act—to take control—in accordance with their moral beliefs and, in doing so, determine the “how” of approaching moral rights and avoiding moral wrongs. The classic traditions cited earlier, as well as many contemporary moral psychologists, see moral motivation as providing a push or a pull to go beyond basic self-interest, beyond immediate pleasure or pain (Mansbridge, 1990). Through this lens of morality, the motivation to fulfill self-interest needs to be controlled in order to accomplish the actualization of moral behavior. For example, to be courageous in a threatening situation, an individual must control the self-interested desire to avoid pain. To be generous, an individual may need to control the self-interested desire to approach a personal pleasure and instead give her money away. In fact, research has shown that when one does not perceive oneself as responsible for (i.e., having control over) one’s own behavior, immoral behavior becomes more likely (Vohs and Schooler, 2008).

The non-human example of the rats responding to a noxious stimulus above contains a parallel—enduring an aversive stimulus in the short term in order to approach security. Human beings accomplish this motivational constraint on a grander scale through a sense of becoming (Higgins, 2005). Human beings are capable of seeing their singular actions or inactions, their approaches and avoidances, as instantiations of a larger long-term project of either moral maintenance or moral growth. In this way, human beings are able to actively act or inhibit their behavior in order to close the gap or maintain the concordance between their actual selves and their ideal- or ought-selves (Higgins, 2005). It is in this way that “what” you are approaching in morality or “why” you are approaching it are only part of the question—“how” you approach it matters critically as well. Morality needs to be pursued across time—sometimes a long time—and matching that controlled achievement to particular goals is a key to successful “ethical becoming.” A common maxim for this everyday phenomenon is, “Life is a journey, not a destination.”

Evidence for control motivation is evident across psychology, from self-determination theory (Deci, 1980; Deci and Ryan, 1985) to self-efficacy theory (Bandura, 1982, 1997). With respect to moral psychology, let’s consider an example of the importance of “how” you do something in a study on charitable giving. Participants were provided with a prompt to go about their decision making in a particular way: either in a way that sees charitable giving as being related to fulfilling an ought *duty*, a goal that must be vigilantly maintained across time; or as being related to an ideal aspiration of *virtue*, a goal that must be eagerly attained across time. For each of these ways of giving to charity, when the way “fit” participants’ particular regulatory focus—the ought duty way fitting with prevention; the ideal aspiration way fitting with promotion—there were significantly higher levels of giving among participants (Cornwell et al., unpublished manuscript). This increase occurred because when the manner of actually going about the decision-making process (the “how”) fit with the particular goals of the individual (the “why”), which made the prospect of giving charity more motivating, in accordance with regulatory fit theory (Higgins, 2000).

Emotional experiences also provide feedback on how well one is managing one’s relation to promotion and prevention goals across multiple situations (Higgins, 2001)—not just “how do I achieve my goal?” but “how am I doing?” This experience also provides a basis for control. For example, if someone fails to maintain a basic moral standard regarding a duty or responsibility, he or she may feel “guilty,” which can increase motivation to try harder to maintain those standards. Similarly, if someone succeeds at fulfilling an ideal moral standard regarding an aspiration, he or she may feel “virtuous,” which can increase motivation to continue to strive for more excellence in the future. The former emotion, being a negative agitation-related emotion, is a prevention focus failure emotion. The latter emotion, being a positive cheerfulness-related emotion, is a promotion focus success emotion (Higgins, 2001). Each of them provides specific feedback that affects future control motivation.

Importantly, the emotions are specifically relevant to the regulatory focus in question, and track the emotional experiences that provide for the most control. Feelings of failure are a “fit” for the prevention focus because they strengthen the vigilance that sustains prevention, leading to greater engagement. In contrast, feelings of success are a “fit” for the promotion focus because they strengthen the eagerness that sustains promotion, leading to greater engagement (Higgins, 2006). If moral emotions are relevant to the “how” of approach/avoidance, i.e., are relevant to control motivation, then we should see that moral and immoral behavior results in those emotions that fit a person’s self-regulatory goals (promotion or prevention) because fit strengthens the engagement that contributes to more effective control. If emotions are not related to control, but instead simply represent positive and negative feedback for good and bad behavior—as a purely hedonic perspective might predict—then the type of emotional feedback should be unrelated to regulatory focus predominance.

These alternative perspectives were tested in another study on charitable giving conducted by Cornwell et al. (unpublished manuscript). Participants were again given the opportunity to donate some of their participant earnings to charity. After their decision, they were asked to report on their internal emotional experiences. Those who were predominantly promotion-focused (vs. prevention-focused) reported differences in how virtuous they felt (low virtue if they didn’t give; high virtue if they did). In contrast, those who were more predominantly prevention-focused (vs. promotion-focused) reported differences in how guilty they felt (high guilt if they didn’t give; low guilt if they did). The positive or negative emotional feedback (experienced differently depending on which type of regulatory focus goal the participant was pursuing) is related to the ongoing ethical project to motivate future action. By experiencing emotional control feedback that matches the “why” of the moral goals, individuals are more engaged and, thus, more able to engage effectively in control over themselves for the sake of their moral standards.

An interesting aspect of these studies of charitable giving is that they not only highlight the importance of the “how” for ethical motivation, but also suggest that the “how” and the “why” can *work together* (i.e., fit) to achieve the most ethical behavior, over and above the simple additive effect of each motivational element in isolation (Higgins, 2006). According to our model, if any of the fundamental aspects of motivation is lacking—the “what,” “how,” or “why”—the effectiveness of an individual’s activity, whether it be an approach or avoidance activity, will be considerably diminished.

## EFFECTIVENESS OF MOTIVE ORGANIZATION

**FIGURE 2:**
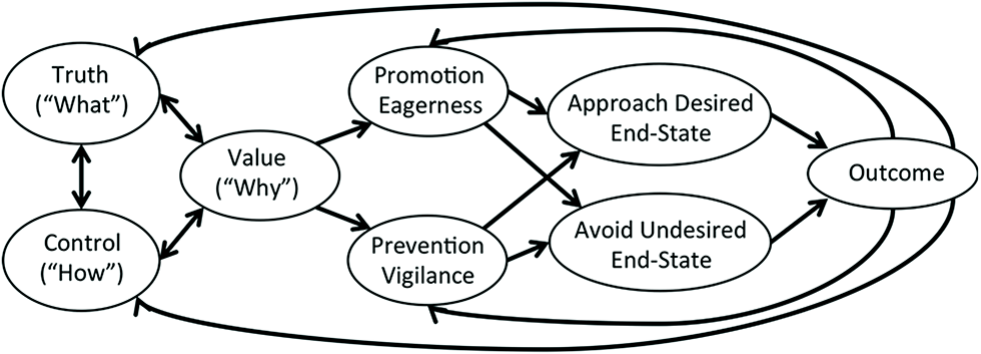
**How the different motivations work together to produce effective approach and avoidance behavior.** Humans and other animals seek to understand the world as it actually is (truth), and the ways in which it relates to and affects the world (control). They determine the goals toward which their actions intend to move (value), which interact with the animal’s understanding of the world (truth) and how their actions affect it (control). When these align in a satisfactory fashion, strategic eagerness or strategic vigilance is instigated leading to the achievement of desirable end-states and the avoidance of undesirable end-states. The consequences of these choices then produces feedback to the animal about whether it understood the world correctly (truth), managed to have the effect wanted (control), and ended up with the desired results (value). Goals are then reevaluated in light of this feedback, and the process begins again. When all of the motives fit with one another, the activity produced will “feel right.”

Each of these kinds of motivations (value, truth, and control) can be regarded as conceptually independent of one another—providing additional motivational grounding for approach and avoidance motivations. Importantly, however, they also need to work together in order that approach and avoidance behaviors are pursued effectively (how the different motivations interact is illustrated in **Figure 2**). An implication of this conceptualization—alluded to above—is that this interactive process underlying approach and avoidance behavior and motivation can occur more or less effectively in a particular individual.

To understand the principles behind this effectiveness of motive organization, it may be useful here to lay out how it develops in humans. To reveal the central importance of organization, examining the independent development of truth, control, and value motivation in isolation is insufficient. Instead, a holistic, integrative perspective is required to reveal how truth, control, and value follow a developmental progression that results in their working together effectively. In the next section, we examine how this development occurs in human psychology to further highlight how motives of truth, control, and value are central to the understanding of approach and avoidance (see also Higgins, 2012).

### DEVELOPMENT OF MOTIVES

Many developmental psychologists who have worked to integrate Piagetian developmental models with new research in cognitive psychology continue to view the transition from infancy into childhood as involving different stages of development. A review of these stages reveals that infants and young children tend to emphasize truth and control effectiveness at least as much as valued outcomes. Moreover, examining these stages in light of similar developmental work by Erikson (1980) and Freud (1927/2011) further argue against a simple division of motivation into approach and avoidance.

According to Erikson (1980), human beings proceed through different phases of “conflict” during which they achieve particular capacities with which to engage with the world in an effective way. The earliest phase, occurring between birth and 2 years of age, is the conflict between trust and mistrust (the oral-sensory period), in which children attain the ability to distinguish between reality (i.e., truth) and fantasy with the help of their caregivers. This stage is primarily related to the desire to understand how the world works, and is achieved through understanding how other human beings in their environment understand the world by sharing in their sense of reality (Erikson, 1980). Children have some basic outcome needs at this stage (e.g., warmth and nourishment), but these needs are not the same as desires for *particular* outcome goals, which develop much later. In many ways, the fulfillment of these basic needs via caregivers serves not just to achieve the outcome, but also to achieve relational motives, which are in many ways connected to epistemic motives, particularly with respect to understanding reality (Hardin and Higgins, 1996). This overall view is consistent with Piagetian and neo-Piagetian perspectives in which the first developmental stage is the sensorimotor stage, when the child learns about its truth and control relationship with the world (Piaget, 1983).

The next phase of development for Erikson (1980) occurs around two to 4 years of age, and is understood as the conflict between autonomy vs. shame and doubt (the muscular-anal period). This stage is the period in which children learn how to control their own behavior and manipulate themselves relative to the world (control). It is also the stage in which children are able to mentally represent objects in their minds not immediately present to their senses (Erikson, 1980). These changes offer children the capacity to reflect on how their actions influence the world and make it change based upon their actions. They also begin to think about how the world might be or might become, not simply how it is. Thus children are motivated in this stage to effectively develop the ability to control aspects of the world within their range of influence.

The third phase of development according to Erikson (1980) is represented by the conflict between initiative and guilt (locomotor-genital), which occurs around the ages of four and five. It is during this stage that children begin to integrate their desires and needs with their understanding of the world and their abilities to act upon it. In other words, during this stage, children begin to have mental representations of goals associated with significant others’ viewpoints on them (Erikson, 1980; Higgins, 1989, 1991). It is also worth noting that during this latter portion of the pre-operational stage, Piaget (1983) noted the development of what he called intuitive thought—the phase during which children begin to ask “why?” During this phase of development (roughly equivalent to Freud’s Oedipal stage), children also become capable of inferring the inner states of others, including what others want the child to become (ideals and oughts). This progress is an essential development in children’s control capacity because children can now take into account others feelings about them, beliefs about them, and goals for them when making choices about what to do or not do (Higgins, 1989, 1991).

It is following this final stage that the effectiveness in the three motivational domains can finally be integrated into a full-fledged system of goal pursuit. At this age, children are finally able to achieve a structural integration of the “what,” “how,” and “why” of behavior. Importantly, this classic developmental work indicates that the motive for truth and control are developmentally foundational for a full-fledged approach/avoidance goal system. It also suggests that those individuals who are most effective at integrating these value, truth, and control motivations to form an integrated whole would experience the most effective approach and avoidance. We now review some recent research in moral psychology supporting this view and note the theoretical possibilities of extending this research to animal welfare science.

### ORGANIZATION OF MOTIVES IN MORAL PSYCHOLOGY

As argued above, the “what,” “how,” and “why” questions of approach and avoidance are of fundamental importance in considering questions of right and wrong, good and bad. The preceding developmental account suggests that the most moral human beings would be those that have effectively organized these motivations to ethical ends—those for whom the questions of “what,” “how,” and “why” all flow *together* into ethical activity. The opinions and reasoning of philosophical and religious thinkers converge on this matter. For them, human beings are not simply the sum total of their behaviors, but have a point of unity about which a judgment can be made: a character or soul—in this case, the animating principle of life (in Greek, *psyche*, in Latin, *anima*), rather than a Cartesian “ghost in the machine.” Those in ancient and classical traditions tend to divide the human soul into three parts: the affective, the volitional, and the rational, typically denoted as the affections, will, and reason, which, in turn, correspond to the motivational constructs of value, control, and truth, respectively. As early as Plato (1992), the best human soul would be one in which the three different aspects worked together for the good of the person (Crombie, 1962). For these thinkers, this working together of the soul constitutes the “good life” and has two major implications for moral psychology.

First, it suggests that that those in whom the three forms of motivation work together would also be the most likely to engage in behavior generally deemed to be ethical. Preliminary evidence supports this hypothesis. We have developed a scale to measure the degree of relational integrity among the three motivations, called the “Effectiveness of Motive Organization” scale (EMO; Cornwell et al., unpublished manuscript). This scale correlates not only with higher effectiveness in each of the three kinds of motivation separately (higher measured truth, control, and value effectiveness; Franks, 2012), but also with lower levels of variance among the independent motives. In other words, the people who score highest on the EMO scale also have the most integrated (i.e., equally high) levels of truth, control, and value effectiveness, suggesting greater mutual support and the absence of a dominant or deficient motivation.

Importantly for the research on ethics, the EMO scale was correlated with Benevolence values over and above other values as measured by the Schwartz Value Inventory (over and above other values such as Achievement or Stimulation; Schwartz, 1992), which are theoretically associated with self-transcendence and altruism. Moreover, scores on the EMO scale significantly predicted the likelihood that an individual will engage in charitable giving in the 4 weeks after measurement, the frequency of self-reported altruistic behaviors, and the likelihood of helping in an experimentally created ambiguous situation (Cornwell et al., unpublished manuscript).

The second implication stemming from the link between the “good life” and an effective motive organization is that the most effective means by which to achieve this integration of motivations should occur at the person level rather than the behavioral level—that is, at the level of moral character rather than moral behavior. An implication of the earlier point regarding the development and integration of the three forms of motivation is that *all* of the motivations are implicated in every behavior an individual engages in or inhibits. Thus, a person’s moral character (i.e., how likely he or she is to engage in moral behavior or inhibit immoral behavior) may be directly related to the *integrity* of his or her motives. This level of analysis most fruitfully occurs at the level of the individual as a whole, rather than the particulars of any given behavior. Notably, the word “integrity” itself refers both to having united (integrated) characteristics *and* a strong moral character.

In the moral psychology literature, there is growing evidence that the construct of moral character is of paramount importance, even though early attempts by trait theorists to measure it empirically were largely regarded as failures (Hartshorne et al., 1930). Researchers have theorized that incorporating judgments of character into our theories of moral judgment would greatly improve their predictive capacity and perhaps help us to understand otherwise puzzling judgments and behavior (Pizarro and Tannenbaum, 2011). Empirical work on the subject has also shown that when making judgments, individuals often judge whether a behavior is *the sort of thing a good person would do* rather than simply judging it according to its negative consequences or conformity to universal rules or norms (Inbar et al., 2011). Furthermore, recent work has shown that judgments of moral character actually predominate over other important dimensions of social judgment (Goodwin et al., 2014). Research has also demonstrated the importance of virtues and character strengths in understanding behavior and success (Peterson and Seligman, 2004). Finally, research has shown that encouraging moral behavior among young children is most effective when their character is commended for performing a particular altruistic behavior as opposed to rewarding them (Grusec and Redler, 1980).

These last results are of particular interest given the earlier developmental account. They suggest that it is only after the development of each of the independent motivations (truth, control, and value) and their integration into a relational whole can a truly “moral” human being come about. In light of this formulation, it is interesting to note that Freud (1927/2011) himself argued that the developmental stage immediately following the locomotor-genital stage (after which each of the three motives is present) is that in which conscience develops. The empirical research cited above on the advantage of praising moral character (vs. rewarding moral behavior) shows that praise during an earlier stage of development is ineffective. The study found that among 5-year-olds, subsequent behavior did not differ as a function of whether their character was praised for their altruistic behavior or they were rewarded for it. However, among 8-year-olds, praising the character of children *did* increase subsequent altruism, whereas reward did not. This difference in the efficacy of character praise vs. reward is consistent with the view that moral character presupposes the ability to organize these motivations in an effective way—children did not respond to praise until age 8, which is after the proposed developmental account above is complete (Grusec and Redler, 1980).

Thus we see how the three kinds of fundamental motivations and their effective organization are critically important for investigating the domain of ethics. Given the centrality of this domain to the lives of so many people, its fundamental nature is apparent. Yet it remains to be seen whether the organization of these different motives is important for non-human animals as well. In the final section, we discuss some of the research suggesting that this question should be answered in the affirmative.

### ORGANIZATION OF MOTIVES IN NON-HUMAN ANIMALS

Though there is a strong philosophical foundation for linking the three forms of motivations and their effective organization to the discipline of ethics, the relation to non-human animals is not as obvious. Non-human animals may have certain characteristics that cause them to behave in ways that we might understand as a kind of precursor to the comprehensive ethical systems found in humans (e.g., Flack and De Waal, 2000). Nevertheless, they may not have certain fundamentally human capacities of consciousness nor take into account the inner states of others the way that humans do (Higgins, 2005), and thus they would not organize themselves according to morality in the same manner. However, by adopting the idea of the “soul” as an integrative animating principle rather than a “rational” ghost in the machine, the same principle could be applied to non-human animals. Indeed, many pre-Enlightenment thinkers, though acknowledging the different capacities of human beings relative to other animal species, nevertheless attribute souls to non-human animals (e.g., Aristotle, 1986). Thus, if the three motivations have measurable outcomes in humans in the domain of ethics, there may be analogous outcomes for an effective motive organization in non-human animals.

The area in which this concept may be of particular interest is in the field of animal welfare science. In addition to moral values and behaviors, the EMO scale is also highly correlated to various measures of well-being (Cornwell et al., unpublished manuscript), such as life satisfaction (Diener et al., 1985) and the perception of one’s life as meaningful (Baumeister et al., 2013). These two relations closely mirror “pleasure” and “meaning” in the pleasure-meaning-engagement triad of human happiness outlined by Peterson et al. (2005; see also Haidt, 2006), and there is other evidence that the EMO scale is related to engagement as well. Thus, the “good life” may be “good” in two senses: “good” as being morally good *and* “good” as being well. Since the latter form of “good” is something that human and non-human animals share, we predict that those animals with the most effective motives organization are the ones that have the best welfare. Indeed, this hypothesis was already implicated in our theoretical exploration of each of these motivations in the context of non-human animals.

While no research to date has directly tackled the question of how motive organization relates to welfare, there are several lines of evidence pointing to the utility of this framework (Franks and Higgins, 2012). For this reason, we believe it could be productive to pursue it as a model for future welfare research. For example, the motivation for food—a valuable outcome—is certainly a hallmark of good welfare: loss of appetite is a strong indicator of illness and poor welfare. Nevertheless, an unchecked motivation for food can also be a sign of poor welfare (D’Eath et al., 2009). An individual who is so preoccupied with food (unrestrained value motivation) that it loses interest in changes to its surroundings (diminished truth motivation) or loses the motivation to engage in species-typical activities (diminished control motivation) has a poor organization of motives and is likely to suffer from poor welfare. Similarly, individual animals subjected to learned helplessness experiment conditions learn that they have no control over the outcomes in their life (Maier and Seligman, 1976), which reflects a disorganization of motives that coincides with poor welfare. Thus, we see preliminary evidence that, as in the domain of moral psychology, the relative effectiveness of the three fundamental forms of motivation could potentially be of critical importance to research among non-human animals as well. As we believe that developing these ideas and testing them across species is an important line of inquiry, we hope to see more research in animal welfare science examining the utility of this framework.

## FINAL COMMENTS

Throughout this paper we have argued that the study of approach and avoidance motivation may benefit from the incorporation of additional perspectives on what other motivations it needs to work with (truth and control motivation) and how, as a value motivation, it can be further differentiated (promotion approach/avoidance vs. prevention approach/avoidance). We have discussed three independent motivations that are presupposed by approach/avoidance: namely truth (the “what” of approach/avoidance), control (the “how” of approach/avoidance), and value (the “why” of approach/avoidance), and we have emphasized the importance of considering how they *work together*. We have provided evidence for our perspective by examining these motivational constructs in human moral psychology and non-human animal welfare science. In so doing, we have noted phenomena with which a purely hedonic approach cannot grapple. We concluded by noting that the *organization* of these three kinds of motives may be of central importance to the larger story of approach/avoidance.

For both moral psychology and animal welfare science, attention to the integrity of motives would involve regarding the individual as a whole at the appropriate level of analysis to strive for a complete understanding of how integrity relates to different kinds of effectiveness (moral character for humans and well-being for all animals). This framework is a reconceptualization of motivation that goes beyond the hedonic principle to extend our ability to address the full complexity of human and non-human behavior.

The hedonic principle that animals approach pleasure and avoid pain has provided scientists with substantial explanatory and predictive power. In recent years, however, some of its limitations as a “one size fits all” distinction have become apparent. In this paper, we have discussed the ways that new developments in motivation science have contributed to the growth of the fields of moral psychology and animal welfare science, and the contributions extend beyond these two alone. Understanding the nature of motivation is essential for understanding humans and other animals, and it is critical that this understanding be equipped to answer the fundamental questions of “what,” “how,” and “why” when it comes to approach and avoidance.

## Conflict of Interest Statement

The authors declare that the research was conducted in the absence of any commercial or financial relationships that could be construed as a potential conflict of interest.
